# The Longitudinal Association between Depressive Symptoms and Initiation of Insulin Therapy in People with Type 2 Diabetes in Primary Care

**DOI:** 10.1371/journal.pone.0078865

**Published:** 2013-11-01

**Authors:** Giesje Nefs, Victor J. M. Pop, Johan Denollet, François Pouwer

**Affiliations:** CORPS - Center of Research on Psychology in Somatic diseases, Department of Medical and Clinical Psychology, Tilburg University, Tilburg, The Netherlands; Iran University of Medical Sciences, Iran (Islamic Republic of)

## Abstract

**Objective:**

To examine whether depressive symptoms are associated with time to insulin initiation in insulin-naïve people with type 2 diabetes in primary care.

**Methods:**

1,389 participants completed the Edinburgh Depression Scale (EDS) in 2005 and were followed until: 1) insulin therapy was started, 2) death, 3) an oral antihyperglycemic drug (OAD) prescription gap >1 year, 4) last OAD prescription in 2010 or 5) the end of the study (December 31, 2010). Cox regression analyses were used to determine whether there was a difference in time to insulin initiation between people with a low versus a high depression score at baseline, adjusting for potential demographic and clinical confounders, including HbA_1c_ levels.

**Results:**

The prevalence of depression (EDS≥12) was 12% (n = 168). After a mean follow-up of 1,597±537 days, 253 (18%) participants had started insulin therapy. The rate of insulin initiation did not differ between depressed and non-depressed participants. People with depression were not more likely to start insulin therapy earlier or later than their non-depressed counterparts (HR = 0.98, 95% CI 0.66–1.45), also after adjustment for sex and age (HR = 0.95, 0.64–1.42). The association remained non-significant when individual candidate confounders were added to the age- and sex-adjusted base model.

**Conclusions:**

In the present study, depression was not associated with time to insulin initiation. The hypothesis that depression is associated with delayed initiation of insulin therapy merits more thorough testing, preferably in studies where more information is available about patient-, provider- and health care system factors that may influence the decision to initiate insulin.

## Introduction

Results from the United Kingdom Prospective Diabetes Study (UKPDS) have shown that improved glycemic control can prevent or delay diabetes complications, in particular those of microvascular origin, in people with type 2 diabetes [1], [2]. Since these landmark findings were published, optimal management of blood glucose levels has become one of the top priorities in diabetes care [3]. The management of hyperglycemia in type 2 diabetes often follows a stepwise strategy, starting with diet and exercise recommendations and followed by prescription of oral agents [3], [4], [5]. Partly due to progressive loss of beta-cell function, glycemic control gradually deteriorates over time [6] and pharmacological monotherapy no longer suffices to attain HbA_1c_ target values [7]. Eventually, even treatment with a combination of oral antidiabetics increased to their maximum doses will fail in most people and the majority will require insulin therapy [7]. Approximately 5–10% of people initially treated with oral antihyperglycemic therapy switch to insulin on a yearly basis [8].

Although national and international guidelines and treatment algorithms advocate rapid treatment modifications when target glycemic goals are not achieved or sustained [3], [4], [5], several studies have shown a delay in insulin initiation of up to five years after failure of oral glucose-lowering agents [9], [10]. While provider attitudes and aspects of the health system are likely to be implicated [11], [12], a reluctance to start insulin therapy has been shown to occur in at least one-quarter of insulin-naïve people with type 2 diabetes [13]. This phenomenon is often termed “psychological insulin resistance” and may encompass a range of negative attitudes towards insulin, including weight concerns, needle phobia and the belief that insulin initiation signifies failure to self-manage diabetes [14].

A factor that might be of particular relevance in this context is depression. Comorbid depression is present in approximately 20% of people with type 2 diabetes [15] and is associated with worse quality of life [16], suboptimal glycemic control [17], an increased risk for the development of vascular complications [18], [19] and a higher mortality risk [20]. Several recent studies have suggested that in insulin-naïve people with type 2 diabetes, higher levels of depression are associated with a more negative appraisal of insulin therapy [21], [22], [23]. Depression is often characterized by fatigue or loss of energy, low self-esteem and a diminished ability to think, concentrate or make decisions [24]. These motivational aspects may underlie a general reluctance to start insulin, which in turn could translate into a later initiation of insulin therapy [22]. However, preliminary results from a small Dutch primary care study (n = 152) suggest that people who switch over to insulin therapy due to secondary failure more frequently suffer from depression [25]. Hence, the association between depression and difficulties with diabetes self-care activities [26], and its ensuing impact on glycemic control may be implicated in this process.

To our knowledge, the role of depressive symptoms in the timing of insulin therapy has not been examined in a large-scale study. Therefore, the primary aim of the present study was to establish whether depressive symptoms are associated with time to insulin initiation in a sample of people with type 2 diabetes in primary care. In addition, we sought to identify demographic and clinical confounders of this relation.

## Materials and Methods

### Procedure

The DiaDDZoB (*Dia*betes, *D*epression, Type *D* personality *Z*uid*o*ost-*B*rabant) Study was designed as a prospective cohort study among people with type 2 diabetes in primary care in South-East Brabant, The Netherlands [27]. A total of 2,460 individuals participated in the 2005 baseline assessment, consisting of a nurse-led interview and the completion of a self-report questionnaire. To increase the accuracy of prescription data, record linkage was sought with the PHARMO Database Network [28], a population-based patient-centric data tracking system which started in 1986 that includes high quality and complete information of patient demographics, drug dispensings, hospitalizations, clinical laboratory, pathology and general practitioner information of 3.2 million community-dwelling inhabitants of 65 municipal areas in The Netherlands. As both the DiaDDZoB and PHARMO databases only contain de-identified patient information, record linkage was realized based on the combination of date of birth, sex, first initial, first letter of family name, first letter of marital name (women only) and zip code. This procedure resulted in successful record linkage for 81% (n = 1,982) of the original DiaDDZoB cohort.

### Participants

The present sample (n = 1,389) includes all DiaDDZoB participants for whom linkage with the PHARMO Database Network could be realized, who completed at least nine items of the EDS during the 2005 baseline assessment, and who had not been prescribed insulin in the six-month period leading up to the baseline assessment. These individuals did not differ significantly from the rest of the DiaDDZoB cohort (n = 1,071) with respect to sex, age, educational level, the presence of chronic co-morbidities (other than cardiovascular disease) or body mass index. However, they were somewhat less likely to have a non-Western ethnicity (2% vs. 5%, *p* = 0.001), to be single (23% vs. 29%, *p* = 0.01), or to have a diabetes duration of three years or more (59% vs. 63%, *p* = 0.02). In addition, they had a slightly lower mean HbA_1c_ level (6.7% vs. 6.8%, *p* = 0.003) and were less likely to have a history of cardiovascular disease (33% vs. 39%, *p* = 0.005), in particular arterial disease (22% vs. 27%, *p* = 0.002), or to have microvascular complications (32% vs. 38%, *p* = 0.02), which was mainly driven by differences in the presence of micro- and/or macroalbuminuria (25% vs. 29%, *p* = 0.03). All 1,389 participants were followed from the baseline assessment in 2005 to the date on which insulin was added to the treatment regimen (end point), date of death, date of last OAD prescription in 2010 or, for those without any prescription of oral antihyperglycemic drugs (OADs) during follow-up, the end of the study period (December 31, 2010), whichever occurred first. To correct for the possibility that peoples’ OAD prescriptions might have been (temporarily) transferred to a pharmacy not included in the PHARMO registry, individuals who were initially prescribed OADs but who showed a period of more than twelve months without any OAD prescription or initiation of insulin were censored on the day of the last OAD prescription before the prescription gap. The DiaDDZoB study protocol was approved by the medical research ethics committee of a local hospital, the Máxima Medical Centre in Veldhoven (NL27239.015.09). Written informed consent was obtained from all participants. The PHARMO compliance committee gave permission to establish the link between the DiaDDZoB cohort and the PHARMO Database Network.

### Assessment of depression

Depressive symptoms during the last seven days were assessed using a validated Dutch version of the Edinburgh Depression Scale (EDS) [29]. Originally designed to assess postpartum depression [30], this questionnaire has now been validated in several other (male and female) strata, including people with type 2 diabetes [31]. Total EDS scores are determined by summing the scores of all ten individual items (four-point scale, total score range 0–30), with higher scores indicating higher levels of depressive symptoms. A total score ≥12 is commonly used to identify people with depression [32]. In the present study, the Cronbach’s alpha of the 10-item EDS scale was 0.84. For participants who only completed 9 items, we replaced the missing value with the mean of the remaining items before calculating the total EDS score.

### Antihyperglycemic medication

All dispensed drugs registered in the PHARMO outpatient pharmacy database are coded according to the Anatomical Therapeutic Chemical Classification (www.whocc.no). Baseline hyperglycemia treatment was determined from dispensing records in the six-month period leading up to the baseline assessment and –based on the sample at hand– subdivided into the general categories lifestyle recommendations only, metformin monotherapy, monotherapy with an agent from a different OAD class, combination of metformin and sulfonylurea, other combination of agents from two OAD classes, and combination of metformin with agents from two or three different OAD classes. We did not differentiate between a switch in OAD classes or add-on therapy, but summarized the classes that were used in the six-month period. The first appearance of the A10A* code (insulin and analogues) in the dispensing records was taken to signify the initiation of insulin therapy, irrespective of (dis)continuation of OAD therapy.

### Baseline demographics, medical history and clinical values

Information regarding sex, age, ethnicity ([white] western vs. non-[white] western), educational level (middle/high vs. low), marital status (having a partner vs. being single) and diabetes duration (less than three years vs. three years or more) was obtained during an interview with participants by the primary care practice nurse or was part of the questionnaire booklet that had to be filled in at home. The primary care practice nurse took a medical history, after which all self-reported medical diagnoses were verified through inspection of the medical record. The Diagnostic Centre Eindhoven, a primary care diagnostic institute, provided results from standard care laboratory tests (HbA_1c_ and albumin levels) and physical examinations (body mass index, eye screening). The results from yearly digital fundus photography were available to ascertain retinopathy (no/yes), while albumin level in a random urine sample was used as a proxy for nephropathy [33]. Micro- and macroalbuminuria were defined as urine albumin concentrations 20–200 and >200 mg/l, respectively. Additional medical co-morbidities included cardiovascular disease (myocardial infarction, bypass/angioplasty, stroke and/or arterial disease) and other chronic conditions (kidney disease, asthma/chronic obstructive pulmonary disease [COPD], cancer, arthrosis and/or rheumatoid arthritis).

### Statistical analyses

Baseline differences in demographic and clinical characteristics between people with and without high depressive symptoms were examined using independent samples t-tests for continuous data and X^2^ tests for categorical data. For both groups, time to insulin initiation was visualized by means of Kaplan-Meier curves, using the log-rank test to compare the two survival curves. A univariable Cox regression analysis was used to provide an effect size for the association between depression and time to insulin initiation, reporting the hazard ratio with corresponding 95% confidence interval. The proportional hazards assumption was checked by visual inspection of the Kaplan-Meier survival curves, Cox regression with a time-dependent covariate and the Harrel and Lee test based on the Schoenfeld residuals. To evaluate whether the association between depression and time to insulin initiation was confounded by specific demographic or clinical factors, we first calculated the percentage change in the regression coefficient for depression before and after adjustment for sex, and repeated this procedure for age [34]. In a next step, we constructed a sex- and age-adjusted base model of the association between depression and time to insulin initiation and examined the percentage change in the regression coefficient for depression when individual candidate confounders were introduced to this base model. Meaningful confounding was defined as a more than 10% change in the regression coefficient [34]. Analyses were performed using PASW Statistics 19 (IBM SPSS Statistics, Somers, NY, USA). A *p*-value <0.05 was considered to be statistically significant.

## Results

The total sample consisted of 1,389 participants (50% female), with a mean age of 67±10 years (range 35–91), mostly self-identifying as (white) western. Overall, participants were in relatively good glycemic control (mean HbA_1c_ 6.7%, 49 mmol/mol), and the majority were being treated with lifestyle recommendations only (27%), metformin monotherapy (20%), monotherapy with an agent from a different OAD class (17%; sulfonylurea derivative, thiazolidinedione or repaglinide), or a combination of metformin and sulfonylurea derivative(s) (31%). Co-morbidities were common, with vascular disease and other major (chronic) medical conditions being present in one-third and one-half of all participants, respectively. Twelve percent (n = 168) had an EDS-score ≥12, indicating depression. Compared with those with an EDS-score <12, participants with depression were more likely to be female, to have a non-western ethnicity, a low educational level and no partner. Furthermore, their medical history was more likely to include a diagnosis of a chronic medical condition (other than cardiovascular), in particular asthma/COPD and arthrosis (Table 1).

**Table 1:** Baseline demographic and clinical characteristics (n = 1,389), stratified by EDS total score.

				N	Total	EDS score	EDS score	*P*
				missing		<12	≥12	value
						(n = 1,221)	(n = 168)	
**Demographics**								
	Female sex			0	50% (699/1389)	48% (590/1221)	65% (109/168)	<0.001
	Age, years			0	67±10	67±10	67±10	0.81
	Non-western ethnicity			15	2% (30/1374)	2% (20/1207)	6% (10/167)	<0.001
	Low educational level			65	64% (843/1324)	62% (723/1167)	76% (120/157)	<0.001
	Being single			15	23% (321/1374)	22% (271/1208)	30% (50/166)	0.03
**Medical history**								
	Diabetes duration ≥ 3 years			13	59% (805/1376)	58% (703/1209)	61% (102/167)	0.47
	Cardiovascular disease			24	33% (453/1365)	33% (391/1198)	37% (62/167)	0.25
		Myocardial infarction		27	11% (153/1362)	11% (135/1195)	11% (18/167)	0.84
		Bypass/angioplasty		22	13% (178/1367)	13% (152/1200)	16% (26/167)	0.30
		Stroke		21	7% (94/1368)	7% (79/1200)	9% (15/168)	0.26
		Arterial disease		29	22% (292/1360)	21% (252/1193)	24% (40/167)	0.40
	Microvascular disease			363	32% (332/1026)	32% (286/906)	38% (46/120)	0.14
		Retinopathy		368	5% (46/1021)	4% (38/903)	7% (8/118)	0.21
		Micro- and/or macroalbuminuria		171	25% (301/1218)	24% (261/1072)	27% (40/146)	0.42
	Other chronic condition(s)			17	48% (661/1372)	47% (565/1205)	58% (96/167)	0.01
		Kidney disease		30	3% (46/1359)	3% (40/1192)	4% (6/167)	0.87
		Asthma/COPD		23	12% (168/1366)	11% (137/1199)	19% (31/167)	0.009
		Cancer		27	9% (123/1362)	9% (106/1195)	10% (17/167)	0.58
		Arthrosis		17	32% (440/1372)	31% (372/1205)	41% (68/167)	0.01
		Rheumatoid arthritis		21	7% (95/1368)	7% (78/1200)	10% (17/168)	0.08
**Hyperglycemia treatment**								
	Number of OAD classes			0	1±0.8	1±0.8	1±0.8	0.26
			Lifestyle only	0	27% (368/1389)	27% (329/1221)	23% (39/168)	0.70
			Monotherapy metformin		20% (277/1389)	20% (243/1221)	20% (34/168)	
			Monotherapy other OAD class		17% (238/1389)	17% (208/1221)	18% (30/168)	
			Metformin + SU derivative(s)		31% (424/1389)	31% (373/1221)	30% (51/168)	
			Other combination of two OAD classes		4% (51/1389)	4% (43/1221)	5% (8/168)	
			Metformin + two or three different OAD classes		2% (31/1389)	2% (25/1221)	4% (6/168)	
**Clinical values**								
	HbA_1c_, % (mmol/mol)			21	6.7±0.8 (49±9)	6.7±0.8 (49±9)	6.6±0.8 (49±8)	0.64
	Body Mass Index (kg/m^2^)			105	29±5	29±5	29±5	0.88

Values are mean ± standard deviation, unless otherwise specified; COPD  =  chronic obstructive pulmonary disease; OAD  =  oral antihyperglycemic drug; SU derivative  =  sulfonylurea derivative.

**Figure 1:**
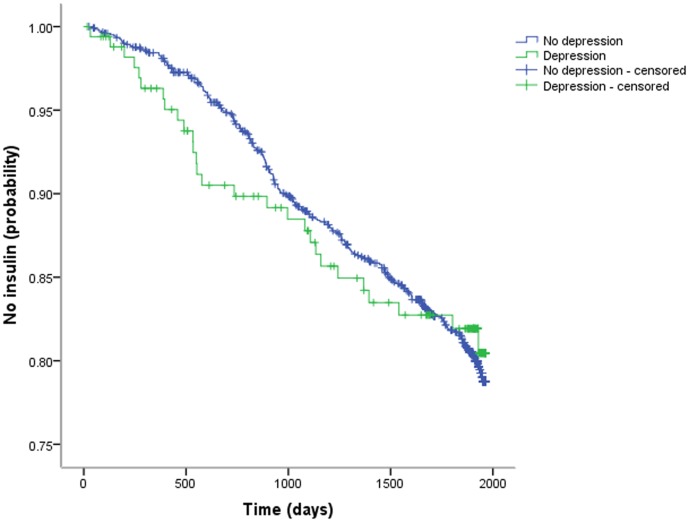
Kaplan-Meier curves EDS score <12 vs. EDS score ≥12.

During a mean follow-up period of 1,597±537 days (range 17–1,964), 253 (18%) participants added insulin to their treatment regimen. The rate of insulin initiation did not differ between people with and without high depressive symptoms (17%, n = 28 vs. 18%, n = 225; *p* = 0.58). When examining the Kaplan-Meier curves, we also did not observe a difference in time to insulin initiation for both groups (log-rank test *X* ^2^ [1] = 0.01, *p* = 0.92), with a mean time to event of 1,783 (95% CI 1,758–1,807) and 1,749 (95% CI 1,671–1,828) days for those without and with depression, respectively (Figure 1). As only 18% of the total sample switched to insulin therapy in the follow-up period, median survival times could not be reported. In univariable Cox regression analysis (n = 1,387; two cases censored before the earliest event in stratum), we found a non-significant HR of insulin initiation (0.98, 95% CI 0.66–1.45, *p* = 0.92).

To check the assumption of proportional hazards, we first examined the Kaplain-Meier curves for people with and without depression (Figure 1). Although the curve for the depression group followed a marginally lower course overall before converging with the non-depressed group at the end of follow-up, both lines appeared to converge briefly at 1000 days. Therefore, we conducted a Cox regression with a time-dependent covariate (splitting the follow-up period in the period before and after 1000 days). When added to the unadjusted regression model, the interaction term of this dichotomous time indicator and depression was not significant (*p* = 0.25), suggesting that the proportional hazard assumption was not violated. The correlation between the Schoenfeld residuals and ranked survival time was significant, but marginal in magnitude (*r* = −0.15, *p* = 0.01).

In the univariable Cox regression analysis, unadjusted confounding could have obscured the association between depression and time to insulin initiation. Therefore, in a next step, potential demographic and clinical confounders were taken into account. Examined separately, sex and age both changed the regression coefficient for depression by more than 10% (Table 2). After simultaneous adjustment for sex and age, the association remained non-significant (HR 0.95, 95% CI 0.64–1.42, *p* = 0.81). With the exception of ethnicity, marital status and body mass index, all demographic and clinical factors that were introduced individually to the sex- and age adjusted base model changed the regression coefficient for depression by more than 10%. For none of the candidate confounders, adjustment produced a statistically significant association between depression and time to insulin initiation.

**Table 2:** Percent change in the regression coefficient for depression in the association with time to insulin initiation, after adjustment for potential confounders.

Potential confounder	N a	*P* value	*P* value	Regression coefficient	Regression coefficient	Change in
		before	after	and HR (95% CI)	and HR (95% CI)	regression
				before adjustment	after adjustment	coefficient (%)
Female sex	1387	0.92	0.86	–0.020; 0.98 (0.66–1.45)	–0.037; 0.96 (0.65–1.43)	85.0%
Age, years	1387	0.92	0.90	–0.020; 0.98 (0.66–1.45)	–0.025; 0.98 (0.66–1.45)	25.0%
Base model (adjusted for sex and age)	1387	0.92	0.81	–0.020; 0.98 (0.66–1.45)	–0.048; 0.95 (0.64–1.42)	140.0%
Base + non–western ethnicity	1372	0.84	0.86	–0.040; 0.96 (0.65–1.43)	–0.037; 0.96 (0.65–1.44)	–7.5%
Base + low education level	1322	0.52	0.46	–0.139; 0.87 (0.57–1.33)	–0.162; 0.85 (0.56–1.30)	16.5%
Base + being single	1372	0.73	0.73	–0.071; 0.93 (0.62–1.39)	–0.072; 0.93 (0.62–1.39)	1.4%
Base + diabetes duration ≥ 3 years	1374	0.88	0.74	–0.030; 0.97 (0.65–1.44)	–0.067; 0.94 (0.63–1.39)	123.3%
Base + cardiovascular disease ^b^	1363	0.83	0.74	–0.044; 0.96 (0.64–1.42)	–0.068; 0.93 (0.63–1.39)	54.5%
Base + microvascular disease ^c^	1024	0.31	0.26	–0.250; 0.78 (0.48–1.27)	–0.283; 0.75 (0.46–1.23)	13.2%
Base + other chronic condition(s) ^d^	1370	0.80	0.75	–0.051; 0.95 (0.64–1.41)	–0.065; 0.94 (0.63–1.39)	27.4%
Base + number of oral antihyperglycemic drug classes	1387	0.81	0.77	–0.048; 0.95 (0.64–1.42)	–0.059; 0.94 (0.63–1.40)	22.9%
Base + HbA_1c_, %	1366	0.83	0.80	–0.044; 0.96 (0.64–1.42)	–0.052; 0.95 (0.64–1.41)	18.2%
Base + Body Mass Index	1282	0.66	0.65	–0.095; 0.91 (0.60–1.38)	–0.096; 0.91 (0.60–1.38)	1.1%

a N varies due to exclusion of cases censored before the earliest event in a stratum (n = 2) and missing values for the candidate confounder at hand; ^b^ Myocardial infarction, bypass/angioplasty, stroke and/or arterial disease; ^c^ Retinopathy and/or micro-/macroalbuminuria; ^d^ Kidney disease, asthma/chronic obstructive pulmonary disease, cancer, arthrosis and/or rheumatoid arthritis.

Similar results were found when examining the association between the continuous EDS total score and time to insulin initiation, with an unadjusted HR of 1.01 (95% CI 0.98–1.03, *p* = 0.68) and a sex- and age adjusted HR of 1.00 (95% CI 0.98–1.03, *p* = 0.81). Except age, ethnicity, marital status and body mass index, all demographic and clinical candidate confounders changed the regression coefficient for depression by more than 10% (range –250% to +75%). In all models the association between depressive symptoms and time to insulin initiation stayed non-significant.

## Discussion

In a sample of 1,389 people with type 2 diabetes, depression (defined as a high level of depressive symptoms) was not associated with an earlier or later start of insulin therapy over a mean follow-up period of 1,597±537 days. After adjustment for potential demographic and clinical confounders, including baseline HbA_1c_ levels, people with depression still did not differ from their non-depressed counterparts with respect to the time before insulin was introduced to their diabetes management.

Although the present study did not find support for an association between depression and treatment intensification with insulin, we believe that further examination of this relation in other samples is warranted, for several reasons. With respect to the sample at hand, relatively few participants (18%) started insulin therapy during the study’s five-and-a-half year follow-up. This was presumably linked to the fact that, at baseline, most participants had HbA_1c_ levels well within the optimal range and one-fourth were managing their blood glucose levels with lifestyle recommendations only. Moreover, in the present sample, few participants (12%) had elevated depression scores [15], [35]. Taking these factors into account, five-and-a-half years of follow-up might not have been long enough to detect any meaningful differences in time to insulin initiation between those with and without depression.

The management of hyperglycemia in type 2 diabetes can be complex for people with diabetes and health care providers alike, as the benefits of optimizing glycemic control through treatment intensification need to be balanced with the needs, preferences and drug tolerances of each individual person [3], [4], [5]. Ultimately, the decision to initiate or refrain from insulin therapy stems from a combination of individual patient and provider factors and characteristics of the health care system, and depression may feed into these processes in a myriad number of ways. If depression systematically leads to an earlier start of insulin therapy in some people but to later insulin commencement in others, these opposite effects may cancel each other out when averaged at the group level. Therefore, it may be hard to characterize the association between depression and insulin initiation, without knowing more about the driving factors behind these treatment decisions. Furthermore, depression itself is a heterogeneous condition, both in terms of severity and subtypes [24]. It is possible that there is a relation between depression and insulin initiation, but only when certain depression characteristics are present, e.g. fatigue or loss of energy, low self-esteem and/or diminished ability to think, concentrate or make decisions.

At least one in four insulin-naïve people with type 2 diabetes is reluctant to start insulin therapy [13] and this process of psychological insulin resistance is associated with the presence of depressive symptoms [21], [22], [23]. Whether there only is a relation with a later start of insulin therapy in those people for whom the motivational aspects of depression cause a general reluctance to start insulin, is yet to be determined in a prospective study. Importantly, these studies should also clarify the context of insulin timing, as a later start can either signify an inappropriate delay of insulin therapy or a longer period of optimal glucose control. Although negative appraisals of insulin therapy do not by definition lead to a delay of insulin therapy, it would be interesting to see if an inappropriate delay of insulin therapy is among the factors that explain why depression is a risk factor for adverse outcomes such as the development of vascular complications and mortality in people with diabetes [19], [20].

Given its close relation with suboptimal glycemic control [17], depression may also be related to an earlier start of insulin therapy. Although time to insulin initiation was not taken into account, a small primary care study (n = 152) suggested that people with type 2 diabetes switching to insulin therapy were 14-times more likely to have co-morbid depression than those individuals who did not start insulin [25]. However, the wide 95% confidence interval around this estimate (2.7–74.9) hints to a relatively small number of individuals with depression in this sample and consequently, this study does not allow any firm inferences about the association between insulin use and depression. We do know, however, that depression is associated with suboptimal medication taking across a range of chronic diseases [36] and that problems with self-management appear to figure prominently in providers’ considerations when choosing antihyperglycemic medications [11], [37], [38]. Perceived problems with self-management on the part of the person with diabetes have been identified as a significant barrier to insulin initiation for health care providers [37], [38], but providers also appear to be more willing to delay insulin initiation if they perceive people with diabetes as more adherent to their medication or appointment regimens [11].

Consultation practices may also play a role in the relation between depression and insulin initiation. Co-morbid depression in diabetes has been associated with increased health care use, including a higher number of ambulatory visits [39]. As a result of this higher contact frequency, physicians may have more opportunities to introduce insulin to the hyperglycemia treatment of depressed people with diabetes. On the other hand, competing demands during care contacts may decrease the likelihood of treatment intensification [40]. In people with diabetes and co-morbid depression, priority might be given to more urgent mood problems.

Recent guidelines and treatment algorithms emphasize the need for early addition of insulin therapy in people who do not meet target goals, in order to reduce the time people are exposed to hyperglycemia [3]. However, previous work suggests that a substantial number of health providers delay insulin therapy until absolutely necessary [11] and there appears to be a tendency to postpone insulin treatment if it is possible to add other oral agents [12]. Of course, there are legitimate clinical reasons to refrain from initiating insulin therapy, including decreased life expectancy and incapacitating comorbidities [3], [4]. Knowing that depression is also more common in people with type 2 diabetes who have co-morbid chronic conditions [41], studies examining the association between depression and insulin initiation should take the potential confounding role of these co-morbid conditions into account. Although we did examine medical history in terms of cardiovascular disease, microvascular complications and the presence of several non-cardiovascular chronic conditions, we were unable to verify the burden of these conditions at baseline.

Several additional study limitations need to be mentioned. First, we used a self-report questionnaire assessing depressive symptoms, while the gold standard for a clinical diagnosis of depression is a structured psychiatric diagnostic interview. In addition, we only focused on baseline depression, while participants’ depression status may have changed over the follow-up period. In a similar vein, by only examining the role of baseline HbA_1c_ levels, we might have missed clinically relevant changes in average blood glucose levels during follow-up that could have shed more light on the association between depression and insulin initiation. Second, the exact reason(s) for insulin initiation were unknown. In some instances insulin is prescribed on a temporary basis, for example in case of corticosteroid use or acute illness such as infections [4]. By focusing solely on community pharmacy dispensings and not including prescriptions from the (inpatient) hospital pharmacy during hospital admissions, we have tried to cancel out some of this confounding. For people who do initiate insulin therapy during a hospital stay as part of their regular hyperglycemia treatment, prescriptions will be transferred to the community pharmacy once they are discharged, thereby introducing only a minor distortion in insulin therapy start date. Third, we cannot rule out that some participants have used insulin in the past. Although we have excluded all people who were prescribed insulin in the six-month period leading up to the baseline assessment, initial insulin therapy may have been withdrawn for some obese individuals due to significant weight loss before that time. Furthermore, insulin may be the first agent prescribed to newly diagnosed individuals with severely uncontrolled diabetes [3], [4]. Fourth, our analyses were based on pharmacy prescriptions, which may not represent actual medication taking. In addition, pharmacy prescriptions only record the start of insulin therapy, and do not provide information about the date on which insulin therapy was first offered to a participant. Fifth, it is unclear whether the introduction of new blood glucose lowering drugs, such as glucagon-like peptide-1 receptor agonists, during follow-up may have influenced insulin initiation. Finally, we cannot rule out that a small minority of our sample have latent auto-immune diabetes of adulthood rather than type 2 diabetes.

Strengths of the study include the large sample of people with type 2 diabetes from a primary care setting, and the detailed medication dispensing data available in the PHARMO Database Network. In The Netherlands, the dispensing records of community pharmacies generally provide an accurate account of all outpatient drug prescriptions. Missing outpatient prescription data (an estimated 5% or less of all outpatient records) are mostly covered by dispensings of antibiotics and analgesics from emergency pharmacies. Furthermore, Dutch community pharmacies dispense the vast majority of outpatient drug prescriptions from both general practitioners and specialists. Therefore, it is unlikely that we would have missed the initiation of insulin therapy due to a referral from primary to secondary care.

In sum, the results of this longitudinal study examining the role of depressive symptoms in the timing of insulin therapy showed that depression was not associated with time to insulin initiation. Additional studies in other samples, preferable incorporating more information about the decision making process that leads to insulin initiation (or not), are needed to further elucidate whether or not depression is associated with initiation of insulin therapy. Given the close relation between depression and negative appraisals of insulin therapy in insulin-naïve people with type 2 diabetes, it is of special clinical interest to examine whether the negative motivational aspects of depression may lead to a delay in insulin initiation. These studies may also explore the role of other psychological or behavioral factors, such as symptoms of anxiety.
